# A traumatic injury leading the lipoma of neck: a rare image

**DOI:** 10.11604/pamj.2022.42.234.36014

**Published:** 2022-07-26

**Authors:** Heena Hussain Pathan, Sanskarsingh Vijaysingh Banafar

**Affiliations:** 1 Department of Community Physiotherapy, Ravi Nair Physiotherapy College, Datta Meghe Institute of Medical Sciences, Wardha, Maharashtra, India,; 2 Department of Musculoskeletal Physiotherapy, Ravi Nair Physiotherapy College, Wardha, Maharashtra, India

**Keywords:** Lipoma, hypertension, benign tumor

## Image in medicine

A 58 years old male visited our hospital with the complaint of swelling over the posterior aspect of the neck after sustaining a trauma as a result of a fall of a cement slab over him 5 years back. The patient is under a combination of metoprolol and telmisartan medication for the rectification of hypertension for 10 years. The patient got admitted to the hospital for the above complaint and has undergone a histopathology report, Magnetic Resonance Imaging (MRI), and Ultrasonography (USG) investigation. In the histopathology report, we received a single container labelled as a resected specimen of large lipoma. On the cut section, a solid, yellowish, homogenous area was identified. MRI investigation reported a lesion over the nape of the neck size measuring from 25x20x8cm in size. USG report stated a lesion over the nape of the neck and occipital region with the dimensions of the left side as 53x25mm approximately, right side as 58x31.5 mm approximately. After both MRI and USG investigation, the patient was diagnosed with lipoma over the nape of the neck and occipital region. The patient had gone through two separate operations in a spare of 11 days. Complete excision of excessive fibrofatty tissue size 10x15 cm lipoma was done. Flap was approximated and romovac drian no 14 placed. Closure was done with ethilon2-0 ac. PPd debridement was done with secondary suturing. Hemostasis was confirmed and patient stable. Shifted to ward and got discharged after 1 week with prescribed medications and physiotherapy exercise.

**Figure 1:**
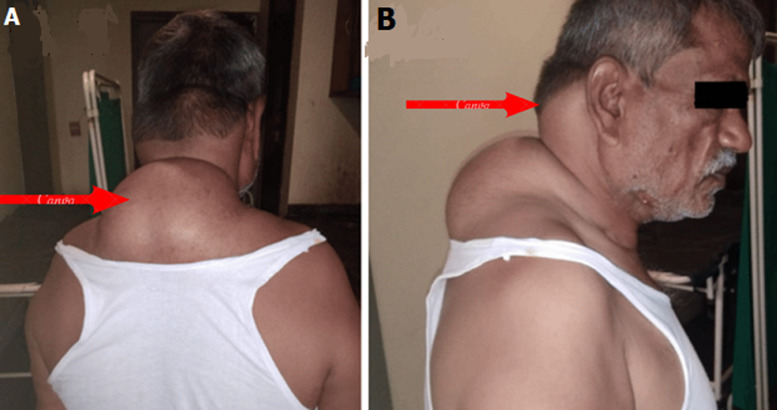
A) dorsal view showing swelling over the nape of the neck; B) lateral view showing swelling over the occipital region

